# Free Cortisol Mediates Associations of Maternal Urinary Heavy Metals with Neonatal Anthropometric Measures: A Cross-Sectional Study

**DOI:** 10.3390/toxics10040167

**Published:** 2022-03-30

**Authors:** Sohyeon Choi, Aram Lee, Gyuyeon Choi, Hyo-Bang Moon, Sungkyoon Kim, Kyungho Choi, Jeongim Park

**Affiliations:** 1 Department of Environmental Health Sciences, Soonchunhyang University, Asan 31538, Korea; sohyeon9535@gmail.com (S.C.); lar2654@naver.com (A.L.); 2 Department of Obstetrics and Gynecology, Soonchunhyang University Hospital, Seoul 04401, Korea; kychoi@schmc.ac.kr; 3 Department of Marine Science and Convergence Technology, Hanyang University, Ansan 15588, Korea; hbmoon@hanyang.ac.kr; 4 Graduate School of Public Health, Seoul National University, Seoul 08826, Korea; ddram2@snu.ac.kr (S.K.); kyungho@snu.ac.kr (K.C.)

**Keywords:** lead, mercury, cadmium, 8-OHdG, free cortisol, Ponderal index, mediation effect

## Abstract

Prenatal exposure to heavy metals is known to be associated with adverse birth outcomes and oxidative stress biomarkers. In this study, we examined whether maternal free cortisol or 8-Hydroxy-2-Deoxyguanosine (8-OHdG) could mediate associations between maternal heavy metal exposure and birth outcomes. A total of 182 healthy pregnant women were recruited. Heavy metals (including Pb, Hg, and Cd), free-cortisol, and 8-OHdG were analyzed in urine at delivery. Birth outcomes including birth weight, length, Ponderal index, and head circumference were measured. To examine associations of maternal urinary heavy metals with biomarkers and birth outcomes, generalized linear models were employed. Birth length was positively associated with Pb (β = 0.78, 95% CI: 0.09–1.46) and Hg (β = 0.84, 95% CI: 0.23–1.45) (both *p* < 0.05). The Ponderal index, a measure of a newborn’s leanness, was negatively associated with maternal urinary Pb (β = −0.23, 95% CI: −0.46–−0.07) and Hg (β = −0.26, 95% CI: −0.44–−0.08) (both *p* < 0.05). No association between maternal Cd and birth outcomes was observed. Most heavy metals showed positive associations with free cortisol and 8-OHdG. Free cortisol was identified as a mediator underlying the observed relationship between Hg and birth length or Ponderal index. This study observed adverse birth outcomes from maternal exposures to Pb and Hg. Increased free cortisol related to Hg exposure was suggested as a possible causal pathway from Hg exposure to birth outcomes such as the Ponderal index.

## 1. Introduction

Prenatal exposure to heavy metals is well-known to be related to adverse health outcomes of fetuses [1,2,3]. Studies have reported associations between maternal heavy metal (such as Pb, Hg, and Cd) exposure and adverse birth anthropometric measures, including birth weight, length, neonatal Ponderal index (a measure of impaired fetal growth), head circumference, preterm birth (PTB), and being small for the gestational age (SGA) [4,5,6].

Heavy metal exposure can lead to an overproduction of reactive oxygen species (ROS) [7,8,9]. ROS induces stressed situations at the cellular level, including structural damage to cells, proteins, nucleic acid, membranes, and lipids, resulting in oxidative DNA damages that may contribute to the pathology of human diseases [8,9,10].

Urinary 8-Hydroxy-2-Deoxyguanosine (8-OHdG) is commonly used as a biomarker of ROS-mediated DNA damage [7,8]. Studies have reported positive relationships between heavy metal exposure and 8-OHdG level [11,12]. Urinary 8-OHdG has been suggested as a good biomarker for oxidative stress related to heavy metal exposure [11,13]. High concentrations of 8-OHdG in maternal urine during pregnancy have been found to be associated with birth anthropometric measures including birth weight, birth length, neonatal Ponderal index, and PTB [14,15,16,17].

Cortisol, the primary stress hormone, is a steroid hormone produced in the adrenal gland following the activation of the hypothalamic-pituitary-adrenal (HPA) axis [18]. It is necessary for survival, influencing important physiological functions such as growth and development. However, a long-lasting secretion of cortisol may cause adverse health effects [19]. A meta-analysis study revealed that maternal cortisol levels are related to preterm birth or lower birth weight and shorter length [20]. Metals including Hg, Pb, and Cd might affect cortisol secretion by altering the HPA physiology, such as glucocorticoid receptor binding or activity that is related to the exertion of glucocorticoids [21]. An animal study also demonstrated a positive association between Hg exposure and free cortisol [22].

Previously, we examined heavy metal exposure in pregnant women, exposure sources, and health effects on their children from the Children’s Health and Environmental Chemicals in Korea (CHECK) cohort [23,24,25,26]. Only a few studies have investigated oxidative DNA damage (8-OHdG) in the pathway between maternal exposure to heavy metals and birth outcomes [11,12,13], and none have assessed the mediation of heavy metal exposure on birth outcomes through the stress hormone (cortisol). Thus, the objective of this study was to explore the mediating roles of 8-OHdG and free cortisol in the association between heavy metals (Pb, Hg, and Cd) and neonatal anthropometric measures such as birth weight, length, head circumference, and Ponderal index.

## 2. Subjects and Methods

### 2.1. Study Population

**Figure 1:**
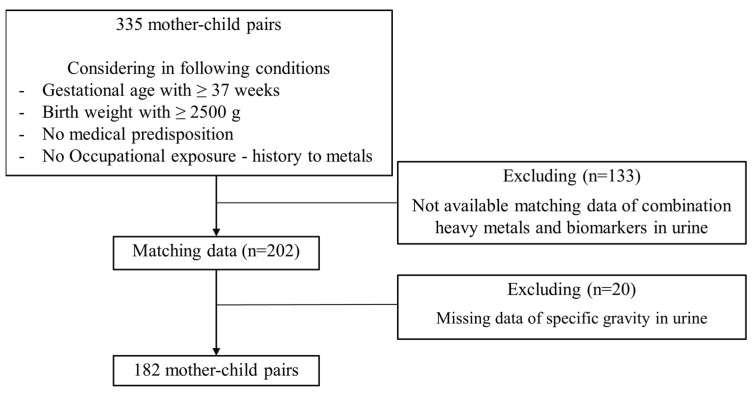
Flowchart showing the selection of the study population.

In the stage of sampling, a total of 335 mother–child pairs in the CHECK cohort were recruited using the following inclusion criteria: gestational age ≥ 37 weeks, birth weight ≥ 2500 g, no medical predisposition, and no occupational exposure history to metals. Among a total of 335 mother-child pairs in the CHECK, 182 pairs were included in the present study after excluding pairs with no matching data for a combination of heavy metals and biomarkers in urine (n = 133) or missing data of specific gravity of urine (n = 20) (Figure 1). This study was conducted after explaining the purpose and methods of this study to participants. It was approved by the IRB (Institutional Review Board) at the School of Public Health, Seoul National University, Korea (IRB no. 8-2012-04-20).

### 2.2. Questionnaries and Birth Data Collection

A systematic questionnaire was used to obtain demographic and pregnancy-related information, including maternal age, pre-pregnancy BMI from maternal height and weight, income, smoking (active and passive) or drinking during pregnancy, delivery mode, and parity. Birth outcomes including sex, birth weight (g), birth length (cm), head circumference (cm), and gestational age at delivery were retrieved from birth records. Ponderal index, an alternative index to BMI, was calculated with the formula of [*birth weight* (*g*)]/[*birth length* (*cm*)]^3^ × 100) [27]. A low Ponderal index of a neonate indicates a disproportionately low birth weight for birth length [28].

### 2.3. Sampling and Urinary Measurement of Heavy Metals, Free Cortisol, and 8-OHdG

Maternal spot urine samples were collected when they arrived at the hospital right before their delivery. All urinary samples were stored at −80 °C until analysis. Pb, Cd, and Hg, the three most frequently reported heavy metals, were analyzed. Pb and Cd were analyzed with graphite furnace atomic absorption spectrometry (PinAAcle 900Z, PerkinElmer). Total Hg was analyzed using Mercury Analyzer (MA-2, Nippon Instrument Corp., Tokyo, Japan). Furthermore, in the quality assurance and quality control processes for measurements of urinary metals, the accuracy and recovery ranges calculated using the spiked urine were 90–110% and 85–100%, respectively. External Quality Assessment Scheme (G-EQUAS, Erlangen, Germany) was also used. Detailed analytical procedures were provided elsewhere [26,29]. Limits of detection for Pb, Cd, and Hg were 1.31 μg/L, 0.08 μg/L, and 0.01 μg/L, respectively. 

Urinary 8-OHdG was measured using a competitive enzyme-linked immunosorbent away with an 8-hydroxy-2-deoxy Guanosine EIA kit (cat no. 589320, Cayman Chemical Company, MI, USA,) [30]. The LOD of urinary 8-OHdG was 33 pg/mL. The urinary free cortisol level was measured using a commercially available chemiluminescence immunoassay or a Unicel DxI 800 Immunoassay System (Beckman Coulter, Brea, CA, USA). The assay range was 0.4–60 μg/dL with an LOD of 0.4 μg/dL. Detailed methods for urinary 8-OHdG and free cortisol were presented previously [31,32]. Specific gravity (SG) of urine was measured using a handheld refractometer (Atago Co., Tokyo, Japan).

### 2.4. Statistical Analyses

Urinary concentrations of Pb, Cd, Hg, free cortisol, and 8-OHdG were adjusted by specific gravity (SG) to compensate for urine dilution [33]. SG has been suggested to be better than creatinine for the adjustment of the urinary dilution effect [34], as pregnancy might influence creatinine metabolism [35]. Metal and biomarker concentrations below LODs were replaced by LOD/√ 2. SG-adjusted levels of urinary Pb, Cd, Hg, free-cortisol, and 8-OHdG were log-transformed to approximate normal distributions. Multiple imputations by chained equations (MICE) were used to impute missing values of demographic characteristics [36]. Missing data for pre-pregnancy BMI (n = 34), smoking during pregnancy (active and passive) (n = 24), drinking during pregnancy (n = 28), and income (n = 28) were imputed using multivariable imputation. Associations between urinary metals and free cortisol or 8-OHdG were examined using the spearman correlation analysis. Effects of metal concentrations on birth outcomes were evaluated using a multiple linear regression model after adjusting covariates. Covariates including maternal age, gestational age (full term pregnancy or not; <39 weeks or ≥39 weeks), infant sex, parity, mode of delivery, pre-pregnancy BMI group (<18.5, 18.5–24.9, >25), smoking (active or passive) or drinking during pregnancy, and monthly household income were considered.

**Figure 2:**
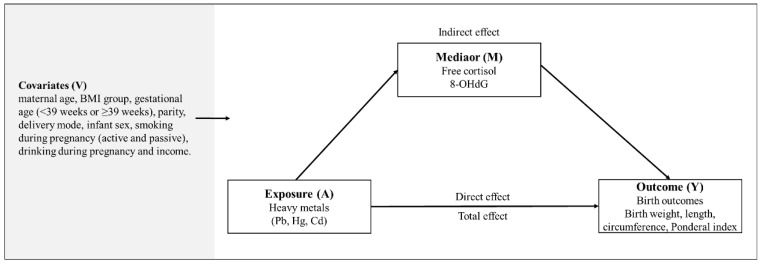
Conceptual model of mediation analysis in the present study.

In order to investigate mediation effects of biomarkers (free-cortisol or 8-OHdG) on the association between heavy metals and birth outcomes, we conducted causal mediation analyses. Unstandardized indirect effects were computed for each of 1000 bootstrapped samples, and 95% confidence intervals were computed by determining indirect effects at the 2.5th and 97.5th percentiles. The total effect was the effect of maternal heavy metal exposure on the outcome. The indirect effect was the effect of heavy metal exposure on birth outcomes mediated through maternal biomarkers. The direct effect was the effect of maternal heavy metal exposure on the birth outcome without maternal biomarkers. The percent mediated was calculated as the value of the indirect effect divided by the sum of the indirect effect and direct effect (total effect). The conceptual model for mediation analysis in this study is illustrated in Figure 2.

Sensitivity analyses were performed to evaluate the robustness of the mediation analysis by considering covariates that might be associated with heavy metal exposure and birth outcomes. In sensitivity analyses, fish and seafood intake were also considered. The intake of fish and seafood was calculated as serving/month and divided into two groups, based on the median, a high intake group or a low intake group. R version 3.6.1 (The Comprehensive R Archive Network: http://cran.r-project.org, accessed 15 March, 2020) was used for all statistical analyses.

## 3. Results

### 3.1. Urinary Concentrations of Heavy Metals, Free-Cortisol, and 8-OHdG

Maternal characteristics and neonatal demographics are presented in Table 1. The mean (±SD) age of mothers was 33.4 ± 3.94 years (range, 23–43 years), and the mean (±SD) length of the pregnancy was 276 ± 7 days. According to pre-pregnancy BMI results, 27.5% (n = 50) of subjects were overweight with a BMI > 25, whereas 15.9% (n = 29) of participants had a BMI < 18.5. The number of participants who had a history of active or passive smoking during pregnancy was 99 (54.4%). Of all neonates, 84 and 98 were boys and girls, respectively. Fifty-three (29.1%) were delivered through C-section. Sixty-nine (37.9%) were the first babies of mothers. The mean (±SD) neonatal weight was 3296 (±337) g. The mean (±SD) neonatal length was 50.1 (±3.2) cm. The mean (±SD) neonatal head circumference was 34.1 (±1.9) cm. The Ponderal index was 2.7 (±0.89) g/cm^3^.

Heavy metals were detected in most urine samples, with detection rates of 91.8%, 99.5%, and 96.7% for Pb, Hg, and Cd, respectively (Table 1). The median concentration (range) was 4.37 (0.92–92.36) μg/L for Pb, 1.25 (0.03–18.3) μg/L for Hg, and 0.72 (0.05–7.80) μg/L for Cd. Maternal urinary Pb and Cd concentrations were found to be different among age groups, with the age group of 40–49 showing the highest (*p* < 0.001) concentrations. Urinary Pb was higher (*p* < 0.03) for mothers who consumed alcohol during pregnancy. Urinary Hg showed a trend of decrease (*p* < 0.05) with increasing pre-pregnancy BMI. Urinary Hg was also significantly different by household income. However, no trend was observed. Smoking during pregnancy and parity did not affect maternal heavy metal concentrations. Concentrations of heavy metals were not associated with gestational age or delivery mode (Table 1). The Spearman correlations between heavy metals are presented in Supplementary Table S1. Moderate or strong correlations between heavy metals were observed (Pb and Hg, r = 0.147, *p* = 0.048; Cd and Hg, r = 0.212, *p* = 0.004).

Free cortisol and 8-OHdG were detected in all maternal urine samples. The median (range) of free cortisol was 34.2 (5.19–178) μg/dL and that of 8-OHdG was 60.5 (29.0–601) ng/mL. Free cortisol and 8-OHdG were moderately correlated (r = 0.148, *p* = 0.046, Supplementary Figure S1). There was no significant difference in free cortisol or 8-OHdG by demographic characteristics (Table 1).

### 3.2. Associations between Urinary Heavy Metals and Free Cortisol or 8-OHdG

Table 2 shows associations between maternal urinary heavy metals and biomarkers, including free cortisol and 8-OHdG. Heavy metal exposures were associated with increased levels of free cortisol and 8-OHdG, except for Pb and free cortisol. These relationships were not altered after adjusting for covariates in statistical models. In covariate adjusted models, maternal urinary free cortisol was positively associated with Hg (β = 0.17, 95% CI: 0.06–0.27, *p* = 0.003) and Cd (β = 0.24, 95% CI: 0.14–0.35, *p* < 0.001), while 8-OHdG was positively associated with Pb (β = 0.18, 95% CI: 0.09–0.26, *p* < 0.001), Hg (β = 0.17, 95% CI: 0.10–0.24, *p* < 0.001), and Cd (β = 0.12, 95% CI: 0.04–0.20, *p* = 0.003).

### 3.3. Associations between Maternal Urinary Metals and Birth Outcomes

**Figure 3:**
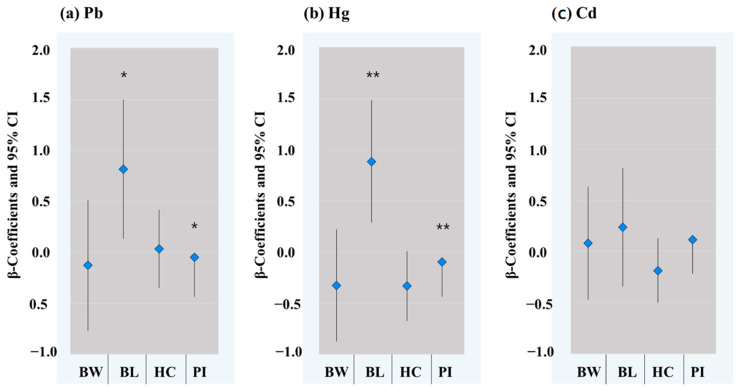
β-coefficients and 95% CIs for correlations of maternal urinary (**a**) Pb, (**b**) Hg, and (**c**) Cd levels with birth weight, birth length, head circumference, and Ponderal index after adjusting for maternal age, pre-pregnancy BMI group, gestational age (<39 weeks or ≥39 weeks), parity, delivery mode, infant sex, smoking during pregnancy (active and passive), drinking during pregnancy, and income. Pb, Cd, and Hg levels in maternal urine samples were adjusted using urine specific gravity. Birth weight was divided by 100 to fit the scale of the figure. * *p* < 0.05, ** *p* < 0.01. BW: birth weight; BL: birth length; HC: head circumference; PI: Ponderal index.

Using multivariable linear regression analyses, a unit increase per Pb or Hg concentration was associated with a 0.82 cm (95% CI; 0.13–1.50, *p* = 0.020) or 0.89 cm (95% CI: 0.29–1.48, *p* = 0.004) increase in birth length, respectively (Figure 3). A unit increase in Pb or Hg concentration was also associated with a −0.25 g/cm^3^ (95% CI: −0.44–−0.05, *p* = 0.014) or −0.27 g/cm^3^ (95% CI: −0.44–−0.10, *p* = 0.002) decrease in the Ponderal index, respectively (Figure 3).

### 3.4. Associations between Levels of Maternal Urinary Biomarkers and Birth Outcomes

Table 3 presents associations between maternal urinary biomarkers and birth outcomes. Multiple linear regression analysis was performed with or without covariate adjustment. Associations were merely affected by the adjustment of covariates. A unit increase in free-cortisol level was associated with a 1.10 cm (95% CI: 0.32–1.88, *p* = 0.006) increase in birth length and a 0.32 g/cm^3^ decrease in the Ponderal index (95% CI: −0.54–−0.10, *p* = 0.005), as shown in results of analysis with covariates adjusted. No significant association between 8-OHdG and birth outcomes was observed. 

### 3.5. Mediation Effect of Biomarkers on the Association between Heavy Metals and Birth Outcomes

**Figure 4:**
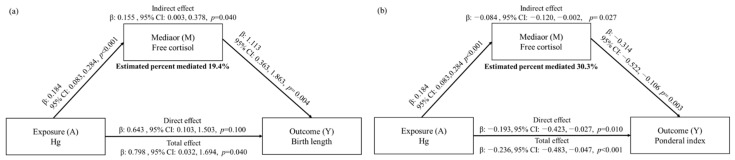
Mediation effect of free cortisol on associations of maternal urinary Hg with (**a**) birth length and (**b**) the Ponderal index. Percent mediated was derived from the indirect effect (β)/(direct effect (β) + indirect effect (β)) × 100.

Mediation analysis was performed to understand the relationship between urinary heavy metals and birth outcomes through biomarkers. Significant mediation effects of free cortisol on birth length (the percent mediated 19.4%) and the Ponderal index (the percent mediated 30.3%) were observed in relation to Hg in maternal urine (Figure 4). Other than Hg-free cortisol-body length and Ponderal index associations, no significant mediation effect of free cortisol was observed for associations between Pb or Cd and birth outcomes. Associations between heavy metals and birth outcomes were not mediated by 8-OHdG (Supplementary Table S3). 

## 4. Discussion

### 4.1. Comparison of Heavy Metals Concentrations in Maternal Urine

Concentrations of maternal urinary Pb, Hg, and Cd in this study were compared with previous studies (Supplementary Table S2). Concentrations of Pb in this study (median: 4.37 μg/L with SG adjustment and 5.95 μg/g creatinine; geometric mean (GM): 4.42 μg/L with SG adjustment and 6.11 μg/g creatinine) were comparable with those of 205 pregnant women in Mexico (median: 3.06 μg/L with SG adjustment; GM: 2.9 μg/L with SG adjustment) [37]. However, Pb concentration in this study was higher than those in pregnant women of Australia (median: 0.7 μg/g creatinine) [38], Spain (median: 3.9 μg/g creatinine) [39], Myanmar (median: 1.8 μg/g creatinine) [40], and China (GM: 1.82 μg/L or 3.19 μg/g creatinine; median: 1.94 μg/L or 2.97 μg/g creatinine) [41]. 

Concentrations of Hg in maternal urine samples of this study (GM: 1.32 μg/L with SG adjustment and 1.80 μg/g creatinine; median: 1.25 μg/L with SG adjustment and 1.70 μg/g creatinine) were slightly lower than those in China (mean: 2.75 μg/L; median: 1.76 μg/L) [42] and Egypt (GM: 7.1 μg/L) [43], but they were not lower than those in Australia (median: < 0.40 μg/L) [38].

Concentrations of Cd (median: 0.72 μg/L with SG adjustment and 0.94 μg/g creatinine; GM: 0.68 μg/L with SG adjustment and 0.94 μg/g creatinine) in this study were slightly higher compared to those in other Asian countries, including Bangladesh (median: 0.58 μg/L) [44], China (GM: 0.64 μg/g creatinine) [45], Japan (GM: 0.77 μg/g creatinine) [46], and Myanmar (median, 0.86 μg/g creatinine) [40]. However, Cd concentrations of pregnant women in Western countries, including Spain (median: 0.54 μg/g creatinine) [39], France (GM: 0.12 μg/L and 0.17 μg/g creatinine) [47], USA (GM: 0.19 μg/L) [48], Mexico (GM: 0.07 μg/L) [37], and Sweden (GM: 0.29 μg/g creatinine) [49], were lower than those in Asian countries, including our study population. Grain including rice is a dominant portion of the standard meal for the majority of people in Asian countries. Rice is one of the major sources of Cd exposure. Higher Cd concentrations in Asian countries might be explained by their high intake of rice as a staple food [50]. 

### 4.2. Association between Heavy Metals and Biomarkers in Maternal Urine Samples

Positive associations of urinary free cortisol and 8-OHdG levels with heavy metals were observed in this study (Table 2), consistent with previous studies [12,51]. Elevated 8-OHdG levels related to heavy metal exposure were consistent with previous studies [12,51]. Heavy metals including Pb, Cd, and Hg can increase the generation of ROS. Elevated ROS could cause adverse effects at the cellular level, including structural damage to cells, proteins, nucleic acids, membranes, and lipids. They could also induce oxidative DNA damages, thus contributing to pathologies of diseases [52]. Urinary 8-OHdG has been reported as a good biomarker for oxidative stress in relation to metal exposures [11,13].

Several studies have reported associations of heavy metals with increased cortisol levels [53,54]. Cortisol, the major human glucocorticoid, is a one of steroid hormones that reflect all stresses. It is secreted by adrenal glands following activation of the hypothalamic-pituitary-adrenal (HPA) axis, a stress-sensitive system [18]. Toxic metals such as Hg can alter the HPA axis function and increase cortisol production [55]. In this study, urinary Hg levels were positively associated with free cortisol levels, consistent with previous studies [21,22,56]. However, some human studies have presented no association between Hg exposure and cortisol [57,58]. 

### 4.3. Maternal Pb Exposure and Birth Outcomes

Increased maternal urinary Pb levels were positively associated with birth lengths of newborns. However, they showed no significant associations with birth weight (Figure 3a). The positive association between Pb and birth length in the present study was contradictory to the results of previous studies [59,60]. One study included a total of 252 mother–infant pairs and found a negative association between cord blood Pb and birth length [59]. Another study suggested that an increment of 1 µg/dl Pb in maternal blood was associated with a decrease of 0.05 cm in birth length [60]. A couple of studies have reported inconsistent associations between maternal Pb exposure and birth weight [61,62]. Some studies have reported negative associations between Pb in maternal blood and birth weight [59,63]. One study observed that the risk of low birth weight was not associated with increased maternal Pb [64]. Sex difference in the association between Pb and birth weight was also observed in a study, showing a positive association for boy infants but no association for girls [62]. 

A negative association was observed for maternal urinary Pb with the Ponderal index of newborns in the present study. The Ponderal index is a good measurement to identify symmetrical or asymmetrical growth status in neonates [28]. The low Ponderal index for neonates may indicate an asymmetrical constitution associated with greater length than weight [28]. The Ponderal index has not been considered in most previous studies, while adverse birth outcomes associated with maternal Pb exposure have been widely presented [59,62,63,64]. A few studies have revealed negative associations between maternal Pb exposures and the Ponderal index [62,64].

### 4.4. Maternal Hg Exposure and Birth Outcomes

Hg exposure showed a positive association with birth length but a negative association with Ponderal index in the present study. Its associations with body weight and head circumstances tended to be negative, although such associations were not statistically significant (Figure 3b). Increased birth length with maternal Hg exposure has been observed in previous studies that analyzed Hg in cord blood [65] or maternal blood at the third trimester [66]. However, a meta-analysis revealed that most studies observed no strong evidence of the association of maternal Hg exposure with adverse fetal growth [67]. A lower Ponderal index at birth is known to be associated with later-life health, particularly coronary heart disease [68,69]. Pb and Hg in maternal blood could be associated with a thinner placental wall [70]. Placenta thickness is an indicator of later intrauterine environmental adequacy for fetal growth [71]. Our observation of negative associations of Pb and Hg with Ponderal index could be explained by an inappropriate uterine environment with a thinner placenta that might be related to heavy metal exposures.

### 4.5. Maternal Cd Exposure and Birth Outcomes

No significant associations were identified between Cd and birth outcomes in this study (Figure 3c), while numerous studies have observed inverse associations between maternal Cd exposure and birth outcomes [5,46,72]. Some previous studies, however, presented no association between Cd in cord blood [61,73] or maternal urinary Cd [74] and birth weight. A non-linear ‘inverted U-shape’ dose response relationship between maternal Cd exposure at 1.5 μg/L and fetal size parameters has been reported [75]. In addition, no association between maternal Cd in urine and birth outcomes in this study was observed after adjusting for weight gain related to birth weight (data not shown).

### 4.6. Free Cortisol and 8-OHdG in Maternal Urine with Anthropometric Measures of Newborns

Negative associations of free cortisol levels with birth circumstance and Ponderal index were observed, whereas free cortisol showed a positive association with birth lengths of newborns (Table 3). It is well-known that maternal cortisol levels are associated with preterm birth [20]. However, studies on the associations of maternal cortisol levels with anthropometric measures of infants have shown inconsistent results [76,77]. Higher cortisol measured in maternal saliva was related to lower birth weights and birth lengths of infants [78]. Regarding the Ponderal index, it showed no association with salivary cortisol in one study [79]. However, an animal study presented a lowered Ponderal index after maternal cortisol infusion [80]. 

8-OHdG showed no significant association with any birth outcomes in the present study (Table 3). Excess DNA oxidation due to increased levels of ROS could be linked to birth outcomes of newborns. Studies have presented consistent results that higher 8-OHdG levels in maternal urine samples are associated with lower birth weights of infants [81,82]. On the other hand, a case-control study of 130 cases of preterm birth and 352 controls suggested that the odds ratios for preterm birth were lower with urinary 8-OHdG concentrations in early pregnancy [17].

### 4.7. Strength and Limitations

Studies that examine the association between maternal exposure to Pb, Hg, and Cd and birth outcomes considering the mediation effects of 8-OHdG and free cortisol have been rarely reported. There is a cohort study suggesting that mercury-induced oxidative stress might induce adverse pregnancy outcomes, such as spontaneous abortion, preeclampsia, and lower birth weight in newborns. However, the study did not address the mediation effect of oxidative stress in the association between Hg exposure and negative birth outcomes [43]. To the best of our knowledge, this is the first report presenting the mediation effect of free cortisol in the associations of maternal urinary Hg with birth length and Ponderal index in newborns. 

This cross-sectional study has limitations. Spot urine samples collected at delivery might not accurately reflect free cortisol or 8-OHdG levels over the course of pregnancy. Previous studies have reported that free cortisol and 8-OHdG concentrations are increased as pregnancy progresses [83,84]. In addition, cortisol levels can vary among people [85]. Associations of heavy metal exposure with 8-OHdG level, free cortisol level, and birth outcomes were insignificant in this study. This could be partly explained by the inclusion criteria (i.e., healthy newborns with full-term birth and birth weight above 2500 g) of this study. A small sample size was also a limitation of this study.

## 5. Conclusions

Associations between maternal urinary heavy metals including Pb, Hg, and Cd and the anthropometric outcomes of newborns were assessed in consideration of free-cortisol and 8-OHdG levels in maternal urine. Maternal Pb and Hg levels were associated with increased birth length but decreased Ponderal index. Free cortisol and 8-OHdG levels were elevated with increasing levels of heavy metals in maternal urine. Mediation analysis suggested that maternal exposure to Hg might induce adverse birth outcomes mediated through increased free cortisol. Such mediation effects were not observed for Pb exposure or 8-OHdG. Further studies are necessary to investigate potential mechanisms underlying such associations between heavy metal exposure and birth outcomes. 

## Figures and Tables

**Table 1:** Demographic characteristics of the study population (n = 182) and their urinary specific gravity (SG)-adjusted concentrations of Pb, Hg, Cd, free cortisol, and 8-OHdG.

Variables	N (%) or Mean ± SD	Median (Range)				
		Pb (μg/L)	Hg (μg/L)	Cd (μg/L)	Free cortisol (μg/dL)	8–OHdG (ng/mL)
Total	182 (100)	4.37 (0.92–92.36)	1.25 (0.03–18.3)	0.72 (0.05–7.80)	34.2 (5.19–178)	60.5 (29.0–601)
Detection frequency (%)		91.8	99.5	96.7	100	100
Maternal age (year)	33.4 ± 3.94					
20–29	25 (13.7)	3.82 (0.92–7.20) **	1.78 (0.59–18.3)	0.84 (0.12–3.28) **	38.8 (22.3–89.0)	76.6 (29.0–137)
30–39	147 (80.8)	4.37 (0.92–22.2)	1.24 (0.05–15.9)	0.68 (0.05–7.80)	33.3 (5.19–178)	60.0 (29.0–602)
40–49	10 (5.5)	5.73 (1.64–92.4)	1.30 (0.03–4.75)	1.94 (0.33–6.17)	24.6 (7.50–59.0)	63.0 (38.1–346)
Prepregnancy BMI (kg/m^2^)	21.7 ±3.92					
<18.5	29 (15.9)	4.95 (0.92–22.2)	1.74 (0.48–18.3) *	0.97 (0.09–6.17)	40.5 (6.33–178)	67.5 (38.6–199)
18.5–24.9	103 (56.6)	4.37 (0.92–19.6)	1.24 (0.03–9.14)	0.65 (0.05–7.80)	30.6 (5.19–178)	58.6 (29.0–602)
>25	50 (27.5)	4.19 (0.92–92.4)	1.08 (0.05–14.5)	0.74 (0.05–7.03)	31.5 (7.60–69.2)	63.9 (29.0–346)
Monthly household income (USD)						
<3000	47 (25.8)	4.30 (1.46–22.2)	1.15 (0.03–6.35) *	0.65 (0.05–3.04)	37.7 (7.54–178)	60.4 (29.0–602)
3000–6000	65 (35.7)	4.48 (0.92–19.6)	1.46 (0.47–15.9)	0.68 (0.05–7.80)	30.0 (5.93–178)	60.0 (29.0–200)
≥6000	70 (38.5)	4.20 (0.92–92.4)	1.18 (0.05–18.3)	0.74 (0.05–7.03)	35.3 (5.19–121)	60.9 (29.0–346)
Smoking status (active or passive) during pregnancy						
No	83 (45.6)	4.43 (0.92–17.2)	1.29 (0.13–14.8)	0.62 (0.05–7.80)	31.7 (5.19–178)	63.9 (29.0–602)
Yes	99 (54.4)	4.42 (0.92–92.4)	1.35 (0.03–18.4)	0.73 (0.05–6.17)	33.5 (7.54–178)	66.6 (29.0–346)
Drinking during pregnancy						
No	159 (87.4)	4.31 (0.92–92.4) *	1.29 (0.05–18.3)	0.71 (0.05–7.03)	5.19 (5.19–178)	60.4 (29.0–602)
Yes	23 (12.6)	5.83 (2.27–22.2)	1.11 (0.03–9.14)	0.73 (0.33–7.80)	6.33 (6.33–178)	60.7 (38.1–200)
Gestational period (days)	276 ± 7.14					
<39 weeks	57	4.67 (0.92–17.2)	1.13 (0.05–18.3) *	0.68 (0.05–2.65)	28.9 (5.19–83.1) *	56.5 (29.0–158) *
≥39 weeks	125	4.30 (0.92–92.4)	1.36 (0.03–15.9)	0.76 (0.05–7.80)	37.7 (5.93–178)	62.7 (29.0–601)
Delivery mode						
Vaginal delivery	129 (70.9)	4.29 (0.92–22.2)	1.35 (0.03–15.9)	0.68 (0.05–7.80)	34.6 (5.93–178)	61.0 (29.0–602)
Cesarean-section	53 (29.1)	4.74 (0.92–92.4)	1.16 (0.05–18.3)	0.74 (0.05–3.40)	33.0 (5.19–89.0)	59.5 (29.0–346)
Parity						
0	69	4.14 (0.92–92.4)	1.34 (0.03–18.3)	0.83 (0.09–3.41)	35.6 (7.54–178)	65.1 (33.3–346)
≥1	113	4.48 (0.92–19.6)	1.24 (0.05–15.9)	0.64 (0.05–7.80)	33.6 (5.19–178)	58.6 (29.0–602)

* *p*-values < 0.05, ** *p*-values < 0.001.

**Table 2:** Association between heavy metals and biomarkers in maternal urine samples (n = 182).

Heavy Metals	Free Cortisol				8-OHdG			
	Unadjusted Model		Adjusted Model *		Unadjusted Model		Adjusted Model *	
	β (95% CI)	*p*	β (95% CI)	*p*	β (95% CI)	*p*	β (95% CI)	*p*
Pb	0.11 (−0.02–0.23)	0.093	0.12 (−0.00–0.25)	0.058	0.16 (0.08–0.25)	<0.001	0.18 (0.09–0.26)	<0.001
Hg	0.18 (0.08–0.28)	<0.001	0.17 (0.06–0.27)	0.003	0.16 (0.09–0.22)	<0.001	0.17 (0.10–0.24)	<0.001
Cd	0.25 (0.15–0.36)	<0.001	0.24 (0.14–0.35)	<0.001	0.14 (0.06–0.21)	<0.001	0.12 (0.04–0.20)	0.003

* Adjusted for maternal age, pre-pregnancy BMI, gestational age (<39 weeks or ≥39 weeks), parity, delivery mode, infant sex, smoking during pregnancy (active and passive), drinking during pregnancy, and income. All measurements in maternal urine samples were adjusted with specific gravity to correct urine dilution effects.

**Table 3:** Multiple linear regression coefficients (95% CI) between biomarkers and neonatal anthropometric measures.

Biomarkers		Birth Weight (g)		Birth Length (cm)		Birth Circumference (cm)		Ponderal Index (g/cm^3^)	
		β (95% CI)	*p*	β (95% CI)	*p*	β (95% CI)	*p*	β (95% CI)	*p*
Free cortisol	Unadjusted	1.80 (−76.8–80.4)	0.964	1.11 (0.36–1.86)	0.004	−0.48 (−0.92–−0.03)	0.037	−0.31 (−0.52–−0.11)	0.003
	Adjusted	−0.27 (−75.1–74.6)	0.994	1.10 (0.32–1.88)	0.006	−0.36 (−0.79–0.07)	0.103	−0.32 (−0.54–−0.10)	0.005
8-OHdG	Unadjusted	27.4 (−84.5–139)	0.632	0.75 (−0.33–1.83)	0.175	−0.06 (−0.69–0.58)	0.862	−0.15 (−0.45–0.15)	0.341
	Adjusted	7.79 (−97.5–113)	0.885	0.64 (−0.48–1.75)	0.263	−0.10 (−0.71–0.52)	0.754	−0.15 (−0.46–0.17)	0.370

Adjusted for maternal age, pre-pregnancy BMI group, gestational age (<39 weeks or ≥39 weeks), parity, delivery mode, infant sex, smoking during pregnancy (active and passive), drinking during pregnancy, and income. All measurements in urine were adjusted with urine specific gravity to correct urine dilution effects.

## Data Availability

Data are provided within the article or Supplementary Material.

## Supplementary Materials

The following supporting information can be downloaded at https://www.mdpi.com/article/10.3390/toxics10040167/s1: Figure S1, Spearman correlations between free cortisol and 8-OHdG in maternal urine; Table S1, Spearman correlations between different heavy metals in maternal urine; Table S2, Comparison of metals levels (μg/L) in maternal urine; Table S3, Mediating effects of biomarkers on relations between heavy metals in maternal urine and birth outcomes.
