# Supplementary material for: Free Cortisol Mediates Associations of Maternal Urinary Heavy Metals with Neonatal Anthropometric Measures: A Cross-Sectional Study
Source: Toxics. 2022 Mar 30;10(4):167. doi: 10.3390/toxics10040167 (PMC9032588; doi:10.3390/toxics10040167)
Supplement: Supplementary file 1 [file toxics-10-00167-s001.zip › toxics-1617453-supplementary.pdf]

Supplementary Materials: Free cortisol mediates associations of maternal urinary heavy metals with neonatal anthropometric measures

Sohyeon Choi, Aram Lee, Gyuyeon Choi, Hyo-Bang Moon, Sungkyoon Kim, Kyungho Choi and Jeongim Park

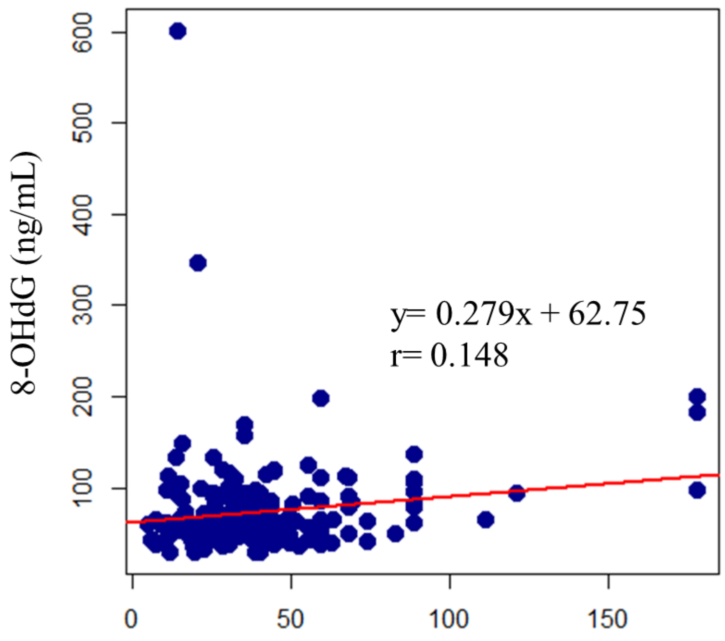

Supplementary Figure S1. Spearman correlations between free cortisol and 8-OHdG in maternal urine.

Supplementary Table S1. Spearman correlations between different heavy metals in maternal urine.

	Pb		Hg	
Parameter	r	*p*	r	*p*
Pb	1			
Hg	0.147	0.048	1	
Cd	0.058	0.436	0.212	0.004

Supplementary Table S2. Comparison of metals levels (μg/L) in maternal urine.

			ug/L				ug/g cret				References
Country	Age	GW +	N	GM	Median	Range	N	GM	Median	Range	
Pb											
South Korea	33	39	202	3.98	4.38	<LOD-14.50	202	6.11	5.94	0.60-155.64	Present study
South Korea¶			182	4.42	4.37	<LOD-92.36					
Australia	32	et al	173		0.55		173		0.7	0.1-7.06	Hinwood AL etal., 2013
China	25	37	205				205	0.48	0.66	<LOD-16.8	Hong Sun et al., 2014
China	28	<37 (n=283)<br>37-38 (n=2,145)<br>>39 (n=4,871)	7,299	1.82	1.94		7,299	3.19	2.97		Cheng L et al., 2017
China	28	34.45					232		3.1		Wang M et al., 2018
China			7,359	1.81				3.69			Wang et al., 2019
Japan							78	0.483	1.19*	0.12-17.4	Shirai et al., 2010
Myanmar	28	38					419		1.8	1.0-3.3	Wai et al., 2017
Spain	>16	3rd trimester					657		3.9		Marta F et al., 2014
Mexico		3rd trimester	205	2.9	3.06	77.5					Ryan CL et al., 2018
Cd											
South Korea	33	39	202	0.61	0.64	<LOD-8.65	202	0.94	0.94	0.07-8.65	Present study
South Korea¶			182	0.68	0.72	<LOD-7.80					
Australia	32		173	0.66*		0.12-2.77	157	0.78*		0.16-4.65	Hinwood AL et al., 2013
Arab	<18		140		5.3	1.9-8.7	140				Karakis IB et al., 2015
Bangladesh	27		212		0.58		212				Karin S. Eet al., 2010
China	18-35	27	5,364				5,364		0.53	0.01-2.85	Yang JE et al., 2016
China			7,359	0.31				0.64			Wang et al., 2019
French	30.3		990	0.12		0.11-0.13	990	0.17			Dereumeaux Cl et al., 2016
Japan							78	0.766	0.976*	<0.04-7.29	Shirai et al., 2010
Myanmar	28	38	419				419	0.5	0.86		Kyi MW et al., 2017
Spain	>16	3rd trimester					657		0.54		Marta F et al., 2014
Sweden	32.5	38.8						0.29		0.1-8.4	Citlalli OY et al., 2018
USA	27.4	3rd trimester	192	0.19	0.18	0.02-17.06					Meghan M et al., 2016
USA	31	24-28	1,388	0.07		0.03–0.15					Alexandra J.W et al., 2018
Hg											
South Korea	33	39	202	1.18	1.25	0.05-21.50	202	1.8	1.7	0.01-21.3	Present study
South Korea¶			182	1.31	1.25	0.03-18.35					
Australia	32		173		<0.40	<0.40-2.31	157		<0.40	<0.40-3.38	Hinwood AL et al., 2013

China	27		418	2.75	418	Jinhua Wu et al., 2014
Egypt	26	3rd trimester	60	7.1*		Aziza EB et al., 2018

+ Gestational age

* Mean

¶ Corrected for SG

Supplementary Table S3. Mediating effects of biomarkers on the relations between heavy metals in maternal urine and birth outcomes

Dependent variables	Independent variables	Indirect effect (95% CI)	*p* value	Direct effect (95% CI)	*p* value	Total effect (95% CI)	*p* value
Mediator: Free cortisol							
Birth weight	Crude						
	Pb	0.467 (-12.0, 12.6)	0.933	-17.7 (-89.6, 49.8)	0.619	-17.3 (-86.3, 48.3)	0.876
	Hg	2.69 (-14.3, 19.5)	0.737	-35.6 (-93.7, 17.7)	0.175	-32.9 (-89.6, 16.2)	0.202
	Cd	-0.509 (-26.3, 22.5)	0.968	8.29 (-49.1, 71.2)	0.774	7.78 (-46.0, 68.2)	0.770
	Model 1[a]						
	Pb	3.60 (-6.08, 19.9)	0.49	-30.5 (-106, 34.5)	0.38	-26.9 (-98.8, 35.2)	0.44
	Hg	5.38 (-6.60, 20.8)	0.45	-32.6 (-96.8, 25.0)	0.29	-27.3 (-90.4, 29.8)	0.38
	Cd	5.64 (-16.7, 28.1)	0.67	17.7 (-41.4, 84.8)	0.53	23.3 (-31.8, 83.5)	0.42
	Model 2[b]						
	Pb	3.50 (-5.87, 20.2)	0.50	-31.1 (-102, 37.6)	0.42	-27.6 (-95.7, 41.6)	0.46
	Hg	5.47 (-6.62, 19.9)	0.37	-32.1 (-91.7, 26.0)	0.33	-26.7 (-85.5, 31.1)	0.41
	Cd	5.54 (-20.2, 31.5)	0.62	19.0 (-48.8, 83.9)	0.59	24.5 (-32.8, 81.2)	0.43
	Model 3[c]						
	Pb	0.244 (-11.6, 13.0)	0.97	-13.3 (-74.8, 46.0)	0.64	-13.0 (-70.0, 42.7)	0.66
	Hg	1.71 (-14.5, 16.0)	0.85	-34.5 (-92.1, 23.6)	0.23	-32.8 (-91.6, 21.8)	0.21
	Cd	-1.04 (-24.7, 19.3)	0.88	8.95 (-49.0, 67.8)	0.69	7.91 (-45.3, 60.9)	0.72
Birth length	Crude						
	Pb	0.104 (-0.039, 0.334)	0.177	0.583 (-0.031, 1.293)	0.065	0.687 (0.028, 1.484)	0.039
	Hg	0.155 (0.003, 0.378)	**0.040**	0.643 (-0.103, 1.503)	0.100	0.798 (0.032, 1.694)	**0.040**
	Cd	0.284 (0.036, 0.619)	0.020	-0.003 (-0.547, 0.558)	0.999	0.281 (-0.201, 0.833)	0.291
	Model 1[a]						
	Pb	0.119 (-0.043, 0.380)	0.170	0.714 (0.082, 1.470)	0.020	0.834 (0.158, 1.670)	0.016
	Hg	0.154 (0.001, 0.410)	**0.046**	0.765 (-0.091, 1.660)	0.086	0.919 (0.020, 1.860)	**0.044**
	Cd	0.312 (0.067, 0.680)	0.008	-0.014 (-0.626, 0.530)	0.880	0.299 (-0.257, 0.850)	0.334
	Model 2[b]						
	Pb	0.116 (-0.047, 0.370)	0.156	0.727 (0.072, 1.480)	0.028	0.843 (0.108, 1.710)	0.022
	Hg	0.156 (-0.005, 0.380)	**0.066**	0.763 (0.003, 1.670)	0.050	0.919 (0.070, 1.850)	**0.034**
	Cd	0.316 (0.064, 0.700)	0.010	-0.011 (-0.622, 0.640)	0.980	0.305 (-0.212, 0.910)	0.250
	Model 3[c]						
	Pb	0.111 (-0.028, 0.369)	0.14	0.704 (0.134, 1.432)	0.022	0.815 (0.184, 1.641)	0.014
	Hg	0.126 (-0.012, 0.355)	**0.09**	0.759 (-0.031, 1.638)	0.070	0.885 (0.044, 1.817)	**0.034**
	Cd	0.270 (0.018, 0.625)	0.03	-0.035 (-0.545, 0.558)	0.922	0.235 (-0.232, 0.791)	0.304

Birth head circumference	Crude						
	Pb	-0.048 (-0.172, 0.020)	0.219	0.023 (-0.423, 0.409)	0.933	-0.026 (-0.500, 0.375)	0.892
	Hg	-0.062 (-0.156, 0.009)	0.096	-0.388 (-1.199, 0.222)	0.424	-0.450 (-1.293, 0.169)	0.326
	Cd	-0.114 (-0.273, 0.004)	0.059	-0.053 (-0.378, 0.257)	0.749	-0.166 (-0.552, 0.162)	0.360
	Model 1[a]						
	Pb	-0.032 (-0.138, 0.030)	0.390	0.027 (-0.371, 0.370)	0.870	-0.005 (-0.402, 0.350)	0.990
	Hg	-0.034 (-0.121, 0.020)	0.310	-0.336 (-1.185, 0.320)	0.570	-0.369 (-1.276, 0.310)	0.550
	Cd	-0.066 (-0.230, 0.050)	0.290	-0.099 (-0.454, 0.200)	0.520	-0.166 (-0.599, 0.170)	0.380
	Model 2[b]						
	Pb	-0.031 (-0.152, 0.030)	0.400	0.028 (-0.338, 0.370)	0.900	-0.003 (-0.392, 0.360)	0.990
	Hg	-0.034 (-0.122, 0.030)	0.310	-0.336 (-1.259, 0.310)	0.530	-0.369 (-1.320, 0.300)	0.500
	Cd	-0.066 (-0.225, 0.050)	0.280	-0.103 (-0.483, 0.190)	0.540	-0.169 (-0.601, 0.150)	0.360
	Model 3[c]						
	Pb	-0.040 (-0.131, 0.018)	0.24	0.073 (-0.303, 0.368)	0.632	0.033 (-0.344, 0.356)	0.822
	Hg	-0.040 (-0.0136, 0.017)	0.21	-1.066 (0.316, 0.600)	-0.333	-0.333 (-1.147, 0.279)	0.572
	Cd	-0.074 (-0.230, 0.024)	0.16	-0.114 (-0.426, 0.150)	0.414	-0.188 (-0.531, 0.113)	0.240
Ponderal	Crude						
index	Pb	-0.029 (-0.100, 0.009)	0.174	-0.202 (-0.441, -0.041)	0.006	-0.231 (-0.514, -0.047)	0.004
	Hg	-0.043 (-0.120, -0.002)	**0.027**	-0.193 (-0.423, -0.027)	0.010	-0.236 (-0.483, -0.047)	**<0.001**
	Cd	-0.084 (-0.213, -0.010)	0.012	0.029 (-0.106, 0.206)	0.759	-0.055 (-0.186, 0.050)	0.336
	Model 1[a]						
	Pb	-0.030 (-0.099, 0.010)	0.142	-0.209 (-0.447, -0.030)	0.012	-0.239 (-0.523, -0.040)	0.008
	Hg	-0.038 (-0.111, -0.0002)	**0.046**	-0.220 (-0.464, -0.039)	0.002	-0.258 (-0.521, -0.061)	**<0.001**
	Cd	-0.085 (-0.227, -0.010)	0.008	0.042 (-0.117, 0.230)	0.686	-0.043 (-0.196, 0.080)	0.486
	Model 2[b]						
	Pb	-0.030 (-0.111, 0.010)	0.210	-0.215 (-0.453, -0.050)	<0.001	-0.244 (-0.537, -0.060)	<0.001
	Hg	-0.039 (-0.112, -0.001)	**0.038**	-0.217 (-0.480, -0.040)	0.004	-0.255 (-0.537, -0.055)	**0.002**
	Cd	-0.086 (-0.204, -0.010)	0.014	0.046 (-0.117, 0.240)	0.706	-0.040 (-0.205, 0.090)	0.524
	Model 3[c]						
	Pb	-0.033 (-0.117, 0.006)	0.135	-0.213 (-0.438, -0.048)	0.006	-0.245 (-0.524, -0.054)	0.005
	Hg	0.037 (-0.119, -0.0002)	**0.046**	-0.232 (-0.476, -0.040)	0.003	-0.268 (-0.541, -0.055)	**<0.001**
	Cd	-0.082 (-0.216, -0.005)	0.032	0.032 (-0.096, 0.207)	0.700	-0.050 (-0.172, 0.048)	0.370
Mediator: 8-OHdG							
Birth weight	Crude						
	Pb	6.25 (-15.7, 29.8)	0.614	-23.5 (-96.2, 51.4)	0.524	-17.3 (-87.5, 51.2)	0.598
	Hg	8.15 (-15.5, 23.6)	0.458	-41.0 (-97.1, 15.4)	0.150	-32.9 (-87.9, 17.0)	0.194
	Cd	3.45 (-19.4, 19.2)	0.742	4.33 (-54.6, 68.9)	0.844	7.78 (-48.6, 66.3)	0.779
	Model 1[a]						

	Pb	9.30 (-10.7, 33.4)	0.37	-22.6 (-98.7, 46.6)	0.53	-13.3 (-82.6, 49.8)	0.71
	Hg	12.7 (-10.6, 32.4)	0.26	-44.4 (-107, 10.3)	0.11	-31.7 (-90.2, 15.4)	0.25
	Cd	4.84 (-15.4, 19.2)	0.60	17.4 (-37.1, 85.1)	0.57	22.2 (-29.7, 84.9)	0.49
	Model 2[b]						
	Pb	9.81 (-11.0, 36.8)	0.42	-24.7 (-96.5, 42.3)	0.49	-14.8 (-80.8, 46.3)	0.66
	Hg	12.8 (-9.37, 31.7)	0.26	-43.6 (-102.9, 14.1)	0.13	-30.9 (-85.3, 21.6)	0.25
	Cd	4.92 (-15.9, 21.3)	0.64	18.8 (-37.7, 85.3)	0.51	23.8 (-33.4, 87.5)	0.39
	Model 3[c]						
	Pb	2.65 (-19.9, 25.0)	0.814	-15.7 (-80.0, 45.4)	0.650	-13.0 (-71.7, 41.7)	0.646
	Hg	5.36 (-16.8, 21.5)	0.596	-38.2 (-98.0, 17.2)	0.208	-32.8 (-88.4, 18.7)	0.242
	Cd	0.55 (-17.5, 13.2)	0.980	7.4 (-43.6, 66.7)	0.746	7.91 (-43.1, 66.5)	0.774
Birth length	Crude						
	Pb	0.078 (-0.121, 0.300)	0.415	0.608 (-0.066, 1.429)	0.083	0.687 (0.045, 1.482)	0.033
	Hg	0.052 (-0.128, 0.192)	0.511	0.746 (0.014, 1.605)	0.045	0.798 (0.043, 1.675)	0.034
	Cd	0.089 (-0.068, 0.299)	0.250	0.193 (-0.290, 0.725)	0.439	0.281 (-0.205, 0.849)	0.281
	Model 1[a]						
	Pb	0.107 (-0.109, 0.340)	0.290	0.658 (-0.096, 1.540)	0.084	0.765 (0.066, 1.560)	0.030
	Hg	0.081 (-0.108, 0.240)	0.354	0.790 (-0.042, 1.670)	0.064	0.872 (0.052, 1.760)	0.032
	Cd	0.103 (-0.028, 0.320)	0.120	0.202 (-0.289, 0.750)	0.420	0.306 (-0.217, 0.870)	0.250
	Model 2[b]						
	Pb	0.109 (-0.094, 0.370)	0.288	0.662 (-0.074, 1.620)	0.096	0.771 (0.071, 1.650)	0.030
	Hg	0.082 (-0.153, 0.270)	0.388	0.790 (-0.010, 1.680)	0.056	0.872 (0.017, 1.780)	0.042
	Cd	0.107 (-0.038, 0.330)	0.140	0.199 (-0.331, 0.760)	0.450	0.306 (-0.216, 0.900)	0.290
	Model 3[c]						
	Pb	0.051 (-0.166, 0.278)	0.586	0.767 (0.071, 1.575)	0.028	0.815 (0.143, 1.631)	0.010
	Hg	0.025 (-0.176, 0.169)	0.718	0.860 (0.049, 1.760)	0.032	0.028 (0.053, 1.815)	0.030
	Cd	0.064 (-0.050, 0.254)	0.302	0.171 (-0.329, 0.681)	0.470	0.235 (-0.251, 0.786)	0.340
Birth head circumference	Crude						
	Pb	0.089 (-0.068, 0.299)	0.250	0.193 (-0.290, 0.725)	0.439	0.281 (-0.205, 0.849)	0.281
	Hg	0.004 (-0.162, 0.120)	0.910	-0.170 (-0.506, 0.112)	0.265	-0.166 (-0.585, 0.156)	0.357
	Cd	0.039 (-0.075, 0.176)	0.497	-0.490 (-1.280, 0.103)	0.193	-0.450 (-1.283, 0.159)	0.309
	Model 1[a]						
	Pb	-0.013 (-0.195, 0.140)	0.900	0.007 (-0.360, 0.350)	0.980	-0.005 (-0.380, 0.350)	0.980
	Hg	0.032 (-0.116, 0.160)	0.600	-0.401 (-1.302, 0.260)	0.500	-0.369 (-1.297, 0.320)	0.580
	Cd	0.001 (-0.159, 0.100)	0.960	-0.166 (-0.499, 0.140)	0.380	-0.165 (-0.578, 0.160)	0.440
	Model 2[b]						
	Pb	-0.015 (-0.237, 0.140)	0.890	0.012 (-0.351, 0.360)	0.950	-0.003 (-0.404, 0.370)	0.990
	Hg	0.031 (-0.121, 0.150)	0.690	-0.401 (-1.251, 0.270)	0.470	-0.369 (-1.233, 0.320)	0.530

	Cd	0.001 (-0.163, 0.100)	0.980	-0.170 (-0.566, 0.150)	0.330	-0.169 (-0.623, 0.180)	0.410
	Model 3[c]						
	Pb	-0.024 (-0.202, 0.114)	0.802	0.056 (-0.294, 0.358)	0.712	0.033 (-0.336, 0.347)	0.826
	Hg	0.018 (-0.102, 0.122)	0.772	-0.351 (-1.146, 0.261)	0.532	-0.333 (-1.155, 0.273)	0.574
	Cd	-0.002 (-0.126, 0.072)	0.964	-0.186 (-0.511, 0.100)	0.232	-0.188 (-0.565, 0.104)	0.272
Ponderal index	Crude						
	Pb	-0.007 (-0.065, 0.047)	0.760	-0.224 (-0.510, -0.036)	0.005	-0.231 (-0.504, -0.048)	0.003
	Hg	-0.002 (-0.033, 0.040)	0.921	-0.234 (-0.468, -0.062)	<0.001	-0.236 (-0.477, -0.059)	0.000
	Cd	-0.017 (-0.073, 0.018)	0.348	-0.037 (-0.150, 0.069)	0.496	-0.055 (-0.187, 0.051)	0.344
	Model 1[a]						
	Pb	-0.008 (-0.074, 0.050)	0.728	-0.231 (-0.523, -0.030)	0.014	-0.239 (-0.515, -0.050)	<0.001
	Hg	0.001 (-0.033, 0.050)	0.948	-0.259 (-0.527, -0.050)	0.002	-0.258 (-0.518, -0.050)	<0.001
	Cd	-0.017 (-0.080, 0.020)	0.350	-0.027 (-0.156, 0.100)	0.660	-0.043 (-0.188, 0.090)	0.520
	Model 2[b]						
	Pb	-0.006 (-0.071, 0.060)	0.808	-0.238 (-0.568, -0.020)	0.012	-0.244 (-0.540, -0.050)	<0.001
	Hg	0.002 (-0.033, 0.070)	0.964	-0.257 (-0.529, -0.050)	0.002	-0.255 (-0.521, -0.050)	<0.001
	Cd	-0.017 (-0.078, 0.020)	0.370	-0.023 (-0.164, 0.110)	0.610	-0.040 (-0.200, 0.100)	0.500
	Model 3[c]						
	Pb	-0.006 (-0.056, 0.053)	0.796	-0.239 (-0.524, -0.039)	0.012	-0.245 (-0.530, -0.052)	0.004
	Hg	0.002 (-0.038, 0.054)	0.920	-0.270 (-0.540, -0.065)	<0.001	-0.268 (-0.542, -0.053)	0.002
	Cd	-0.015 (-0.072, 0.015)	0.358	-0.035 (-0.140, 0.082)	0.500	-0.050 (-0.157, 0.054)	0.326

If both indirect effect and total effect are statistically significant are highlighted (*p*-values < 0.05).

a Multivariate linear regression adjusted for maternal age, BMI group, income, drinking during pregnancy and smoking during pregnancy (active and passive).

b Multivariate linear regression adjusted for Model 1 + fish intake and seafood intake

c Multivariate linear regression adjusted for Model 1 + gestational age (<39 weeks or ≥39 weeks), parity, delivery mode and infant sex.
